# (Methanol-κ*O*)(perchlorato-κ*O*)bis­(triphenyl­phosphine-κ*P*)silver(I)

**DOI:** 10.1107/S160053681002814X

**Published:** 2010-07-21

**Authors:** Li-Na Cui, Qiong-Hua Jin, Ke-Yi Hu, Cun-Lin Zhang

**Affiliations:** a Department of Chemistry, Capital Normal University, Beijing 100048, People’s Republic of China; b Beijing Key Laboratory for Terahertz Spectroscopy and Imaging, Key Laboratory of Terahertz Optoelectronics, Ministry of Education, Capital Normal University, Beijing 100048, People’s Republic of China

## Abstract

In the title complex, [Ag(ClO_4_)(CH_3_OH)(C_18_H_15_P)_2_], the angles around the central Ag^+^ ion indicate that it is in a distorted tetrahedral coordination. The coordination sphere of silver is formed by two P atoms of two triphenyl­phosphine ligands, one O atom of a perchlorate anion and one O atom of a methanol mol­ecule. The crystal structure is stablized by a bifurcated inter­molecular O—H⋯O hydrogen bond, involving the O—H donor from methanol and two acceptor O atoms from the perchlorate anion, so forming a zigzag chain propagating in [010].

## Related literature

For related structures, see: Cui *et al.* (2010 ▶); Cingolani *et al.* (2002 ▶); Nicola *et al.* (2007 ▶); Pettinari *et al.* (2007 ▶); Effendy *et al.* (2007*a* ▶,*b* ▶); Awaleh *et al.* (2005 ▶); Balakrishna *et al.* (2009 ▶). For general backgound to the structural chemistry of silver(I) complexes with ligands containing phosphine groups and nitro­gen atoms, see: Jin *et al.* (2010 ▶); Wu *et al.* (2009 ▶).
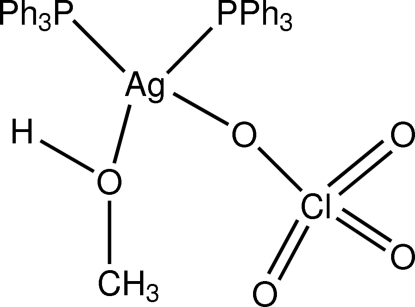


## Experimental

### 

#### Crystal data

[Ag(ClO_4_)(CH_4_O)(C_18_H_15_P)_2_]
*M* *_r_* = 763.90
Monoclinic, 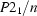

*a* = 13.6426 (15) Å
*b* = 12.8444 (14) Å
*c* = 19.714 (2) Å
β = 92.602 (1)°
*V* = 3450.9 (7) Å^3^
*Z* = 4
Mo *K*α radiation
μ = 0.80 mm^−1^
*T* = 298 K
0.33 × 0.22 × 0.14 mm

#### Data collection

Bruker SMART CCD area-detector diffractometer
Absorption correction: multi-scan (*SADABS*; Bruker, 2007 ▶) *T*_min_ = 0.779, *T*_max_ = 0.897
17113 measured reflections
6073 independent reflections
3802 reflections with *I* > 2σ(*I*)
*R*_int_ = 0.040

#### Refinement

*R*[*F* ^2^ > 2σ(*F* ^2^)] = 0.040
*wR*(*F* ^2^) = 0.103
*S* = 1.08
6073 reflections
415 parameters
H-atom parameters constrained
Δρ_max_ = 0.49 e Å^−3^
Δρ_min_ = −0.32 e Å^−3^

### 

Data collection: *SMART* (Bruker, 2007 ▶); cell refinement: *SAINT-Plus* (Bruker, 2007 ▶); data reduction: *SAINT-Plus*; program(s) used to solve structure: *SHELXS97* (Sheldrick, 2008 ▶); program(s) used to refine structure: *SHELXL97* (Sheldrick, 2008 ▶); molecular graphics: *SHELXTL* (Sheldrick, 2008 ▶); software used to prepare material for publication: *SHELXTL*.

## Supplementary Material

Crystal structure: contains datablocks global, I. DOI: 10.1107/S160053681002814X/su2191sup1.cif

Structure factors: contains datablocks I. DOI: 10.1107/S160053681002814X/su2191Isup2.hkl

Additional supplementary materials:  crystallographic information; 3D view; checkCIF report

## Figures and Tables

**Table 1:** Hydrogen-bond geometry (Å, °)

*D*—H⋯*A*	*D*—H	H⋯*A*	*D*⋯*A*	*D*—H⋯*A*
O5—H5⋯O2^i^	0.82	2.42	3.157 (6)	151
O5—H5⋯O3^i^	0.82	2.30	3.033 (6)	150

Symmetry code: (i) 
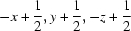
.

**Fig. 1.**
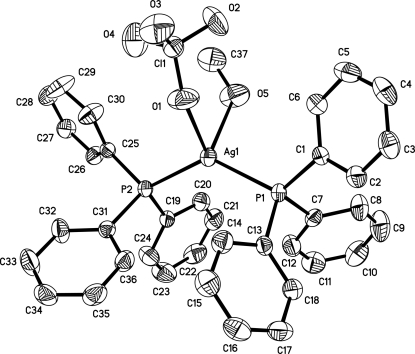
Perspective view of the basic unit of the title complex, (1), with displacement ellipsoids drawn at the 50% probability level. Hydrogen atoms have been omitted for clarity.

## supplementary crystallographic information

### Comment

As a part of our studies on the systematic structural chemistry of silver(I) complexes with ligands containing phosphine and nitrogen atoms (Jin *et al.*, 2010; Wu *et al.*,2009), we synthesized the new title complex, (1), under catalysis of 2-aminopyrimidine. A similar complex ([Ag(PPh_3_)_3_(ClO_4_)] (2) (Cui *et al.*, 2010) was synthesized by the same reaction as for complex (1), but with a longer evaporation time. It can be assumed that during the long period of crystal growth, a PPh_3_ group replaces the coordinated CH_3_OH molecule in (1), resulting in the transformation to complex (2).

The molecular structure of the title complex, (1), is depicted in Fig. 1. The Ag^+^ ion is four coordinated by two phosphorus atoms from the two PPh_3_ ligands, one oxygen atom (O1) from a ClO_4_ anion and one O-atom (O5) from a molecule of methanol. The Ag—P distances of 2.4308 (11) Å and 2.4276 (11) Å are shorter than those observed in complex (2), where the Ag-P distances vary between 2.5047 (13) - 2.5641 (14) Å. They are however longer than those in complexes AgNO_2_:PPh_3_(1:1) [2.3918 (4) Å], and AgNO_2_:PPh_3_(1:2) [2.412 (1)–2.440 (1) Å] (Cingolani *et al.*, 2002). In complex (1) the Ag—O1(perchlorate) distance of 2.540 (4) Å is shorter than that in complex (2) [2.668 (14) Å], and distance Ag—O5(methanol) is 2.414 (4) Å.

In complex (1) the P—Ag—O angles are in the range 97.68 (10) - 114.20 (9) °, the P—Ag—P angle is 133.15 (4) ° and angle O1—Ag—O5 is 92.22 (13) °. This comfirms the distored tetrahedral environment around the silver atom. In complex (2) the P—Ag—O angles are in the range of 87.1 (4) - 118.1 (4) °, while the P—Ag—P angles are in the range of 114.70 (4) - 119.17 (5)°. Other similar complexes include adducts AgX:PPh_3_:*L* where *X* is a simple inorganic or organic anion, including nitrate (Jin *et al.*, 2010; Nicola *et al.*, 2007), nitrite (Pettinari *et al.*,2007), acetate (Effendy *et al.*,2007*a*), perchlorate (Effendy *et al.*,2007*b*), and trifluoroacetate (Awaleh *et al.*, 2005; Balakrishna *et al.*; 2009; Wu *et al.*, 2009).

In the crystal structure of the title complex, (1), symmetry related molecules are linked via a bifocated O-H···O hydrogen bond involving the methanol OH group and two perchlorate O-atoms, O2 and O3 (Table 1). In this manner zigzag chains are formed propagating along [010].

### Experimental

A mixture of AgClO_4_, PPh_3_ and 2-aminopyrimidine, in the molar ratio of 1:1:2, in CH_2_Cl_2_ and MeOH (10 ml,V/V=1/1) was stirred for 2 h at room temperature, then filtered. Subsequent slow evaporation of the filtrate resulted in the formation of colorless crystals of the title complex (1). Crystals, suitable for single-crystal X-ray diffraction, were selected directly from the sample as prepared. Analysis Found (%): C 58.50, H 5.02; calculated: C 58.19, H 4.45.

### Refinement

The H-atoms were included in calculated positions and treated as riding atoms: O-H = 0.82 Å, C-H 0.93 - 0.96 Å with U_iso_(H) = k × U_eq_(parent O or C-atom), where k = 1.5 for OH and CH_3_ H-atoms, and k = 1.2 for all other H-atoms.

### Crystal data

**Table:**

[Ag(ClO_4_)(CH_4_O)(C_18_H_15_P)_2_]	*F*(000) = 1560
*M**_r_* = 763.90	*D*_x_ = 1.470 Mg m^−^^3^
Monoclinic, *P*2_1_/*n*	Mo *K*α radiation, λ = 0.71073 Å
Hall symbol: -P 2yn	Cell parameters from 4281 reflections
*a* = 13.6426 (15) Å	θ = 2.6–23.6°
*b* = 12.8444 (14) Å	µ = 0.80 mm^−^^1^
*c* = 19.714 (2) Å	*T* = 298 K
β = 92.602 (1)°	Block, colourless
*V* = 3450.9 (7) Å^3^	0.33 × 0.22 × 0.14 mm
*Z* = 4	

### Data collection

**Table:**

Bruker SMART CCD area-detector diffractometer	6073 independent reflections
Radiation source: fine-focus sealed tube	3802 reflections with *I* > 2σ(*I*)
graphite	*R*_int_ = 0.040
phi and ω scans	θ_max_ = 25.0°, θ_min_ = 1.8°
Absorption correction: multi-scan (*SADABS*; Bruker, 2007)	*h* = −9→16
*T*_min_ = 0.779, *T*_max_ = 0.897	*k* = −15→13
17113 measured reflections	*l* = −23→19

### Refinement

**Table:**

Refinement on *F*^2^	Primary atom site location: structure-invariant direct methods
Least-squares matrix: full	Secondary atom site location: difference Fourier map
*R*[*F*^2^ > 2σ(*F*^2^)] = 0.040	Hydrogen site location: inferred from neighbouring sites
*wR*(*F*^2^) = 0.103	H-atom parameters constrained
*S* = 1.08	*w* = 1/[σ^2^(*F*_o_^2^) + (0.0349*P*)^2^ + 2.0561*P*] where *P* = (*F*_o_^2^ + 2*F*_c_^2^)/3
6073 reflections	(Δ/σ)_max_ < 0.001
415 parameters	Δρ_max_ = 0.49 e Å^−^^3^
0 restraints	Δρ_min_ = −0.32 e Å^−^^3^

### Special details

**Table:**

Geometry. Bond distances, angles etc. have been calculated using the rounded fractional coordinates. All su's are estimated from the variances of the (full) variance-covariance matrix. The cell esds are taken into account in the estimation of distances, angles and torsion angles
Refinement. Refinement of *F*^2^ against ALL reflections. The weighted *R*-factor *wR* and goodness of fit *S* are based on *F*^2^, conventional *R*-factors *R* are based on *F*, with *F* set to zero for negative *F*^2^. The threshold expression of *F*^2^ > σ(*F*^2^) is used only for calculating *R*-factors(gt) *etc*. and is not relevant to the choice of reflections for refinement. *R*-factors based on *F*^2^ are statistically about twice as large as those based on *F*, and *R*- factors based on ALL data will be even larger.

### Fractional atomic coordinates and isotropic or equivalent isotropic displacement parameters (Å^2^)

**Table:**

	*x*	*y*	*z*	*U*_iso_*/*U*_eq_	
Ag1	0.35069 (2)	0.19066 (3)	0.13302 (2)	0.0492 (1)	
Cl1	0.23718 (9)	−0.06402 (9)	0.15761 (6)	0.0563 (5)	
P1	0.51164 (8)	0.18659 (9)	0.19116 (6)	0.0434 (4)	
P2	0.28652 (8)	0.27792 (9)	0.03118 (6)	0.0402 (4)	
O1	0.3162 (3)	−0.0036 (3)	0.1356 (2)	0.1009 (19)	
O2	0.2245 (3)	−0.0476 (3)	0.2273 (2)	0.104 (2)	
O3	0.2556 (3)	−0.1698 (3)	0.1447 (2)	0.105 (2)	
O4	0.1519 (3)	−0.0337 (4)	0.1199 (2)	0.116 (2)	
O5	0.2320 (4)	0.2257 (3)	0.2172 (2)	0.117 (2)	
C1	0.5365 (3)	0.0933 (3)	0.2592 (2)	0.0441 (17)	
C2	0.6309 (3)	0.0626 (4)	0.2795 (2)	0.0595 (17)	
C3	0.6456 (4)	−0.0130 (4)	0.3280 (3)	0.068 (2)	
C4	0.5684 (5)	−0.0585 (4)	0.3573 (3)	0.072 (2)	
C5	0.4755 (4)	−0.0293 (4)	0.3378 (3)	0.073 (2)	
C6	0.4585 (4)	0.0467 (4)	0.2894 (2)	0.0567 (17)	
C7	0.5424 (3)	0.3148 (3)	0.2245 (2)	0.0461 (16)	
C8	0.5697 (4)	0.3343 (4)	0.2909 (3)	0.071 (2)	
C9	0.5893 (5)	0.4351 (5)	0.3125 (3)	0.097 (3)	
C10	0.5824 (4)	0.5160 (5)	0.2679 (4)	0.084 (3)	
C11	0.5531 (4)	0.4978 (4)	0.2022 (3)	0.079 (3)	
C12	0.5331 (4)	0.3987 (4)	0.1808 (3)	0.065 (2)	
C13	0.6084 (3)	0.1580 (3)	0.1335 (2)	0.0432 (17)	
C14	0.5900 (4)	0.0818 (4)	0.0847 (3)	0.0598 (19)	
C15	0.6607 (4)	0.0530 (4)	0.0408 (3)	0.073 (2)	
C16	0.7508 (4)	0.0993 (4)	0.0451 (3)	0.068 (2)	
C17	0.7709 (4)	0.1730 (4)	0.0933 (3)	0.067 (2)	
C18	0.7004 (3)	0.2023 (4)	0.1375 (2)	0.0563 (17)	
C19	0.3053 (3)	0.4171 (3)	0.0383 (2)	0.0445 (17)	
C20	0.2879 (3)	0.4642 (4)	0.0994 (3)	0.0593 (19)	
C21	0.2911 (4)	0.5714 (4)	0.1062 (3)	0.074 (2)	
C22	0.3139 (4)	0.6310 (4)	0.0518 (4)	0.082 (3)	
C23	0.3348 (5)	0.5859 (4)	−0.0079 (3)	0.090 (3)	
C24	0.3307 (4)	0.4786 (4)	−0.0151 (3)	0.070 (2)	
C25	0.1556 (3)	0.2673 (3)	0.0099 (2)	0.0397 (17)	
C26	0.0983 (3)	0.3488 (4)	−0.0128 (2)	0.0544 (17)	
C27	0.0012 (3)	0.3347 (4)	−0.0322 (3)	0.061 (2)	
C28	−0.0394 (4)	0.2396 (5)	−0.0299 (3)	0.070 (2)	
C29	0.0159 (4)	0.1583 (4)	−0.0061 (4)	0.095 (3)	
C30	0.1122 (4)	0.1719 (4)	0.0146 (3)	0.074 (2)	
C31	0.3441 (3)	0.2409 (3)	−0.0468 (2)	0.0430 (17)	
C32	0.2929 (4)	0.2312 (4)	−0.1076 (3)	0.070 (2)	
C33	0.3389 (5)	0.1985 (5)	−0.1648 (3)	0.086 (3)	
C34	0.4358 (5)	0.1776 (4)	−0.1622 (3)	0.075 (3)	
C35	0.4887 (4)	0.1880 (4)	−0.1021 (3)	0.076 (2)	
C36	0.4426 (3)	0.2193 (4)	−0.0450 (3)	0.063 (2)	
C37	0.1331 (5)	0.2035 (5)	0.2160 (3)	0.100 (3)	
H2	0.68450	0.09350	0.26000	0.0720*	
H3	0.70910	−0.03320	0.34100	0.0810*	
H4	0.57890	−0.10930	0.39040	0.0870*	
H5	0.24420	0.27180	0.24520	0.1760*	
H5A	0.42250	−0.06110	0.35750	0.0880*	
H6	0.39460	0.06650	0.27710	0.0680*	
H8	0.57510	0.27960	0.32170	0.0860*	
H9	0.60730	0.44770	0.35780	0.1170*	
H10	0.59770	0.58330	0.28230	0.1010*	
H11	0.54670	0.55300	0.17180	0.0940*	
H12	0.51280	0.38740	0.13570	0.0780*	
H14	0.52890	0.04960	0.08170	0.0720*	
H15	0.64710	0.00210	0.00820	0.0870*	
H16	0.79850	0.08050	0.01520	0.0810*	
H17	0.83260	0.20380	0.09650	0.0800*	
H18	0.71520	0.25260	0.17040	0.0680*	
H20	0.27380	0.42330	0.13670	0.0710*	
H21	0.27780	0.60250	0.14730	0.0890*	
H22	0.31510	0.70310	0.05580	0.0980*	
H23	0.35200	0.62710	−0.04420	0.1080*	
H24	0.34520	0.44820	−0.05630	0.0840*	
H26	0.12570	0.41490	−0.01510	0.0650*	
H27	−0.03660	0.39130	−0.04710	0.0740*	
H28	−0.10450	0.22950	−0.04450	0.0840*	
H29	−0.01210	0.09240	−0.00390	0.1140*	
H30	0.14840	0.11580	0.03200	0.0880*	
H32	0.22630	0.24680	−0.11050	0.0830*	
H33	0.30260	0.19080	−0.20560	0.1040*	
H34	0.46640	0.15640	−0.20100	0.0900*	
H35	0.55570	0.17390	−0.09980	0.0910*	
H36	0.47910	0.22600	−0.00420	0.0760*	
H37A	0.10040	0.25310	0.24350	0.1490*	
H37B	0.12360	0.13470	0.23350	0.1490*	
H37C	0.10650	0.20730	0.17010	0.1490*	

### Atomic displacement parameters (Å^2^)

**Table:**

	*U*^11^	*U*^22^	*U*^33^	*U*^12^	*U*^13^	*U*^23^
Ag1	0.0433 (2)	0.0591 (2)	0.0444 (2)	−0.0019 (2)	−0.0073 (2)	0.0065 (2)
Cl1	0.0676 (9)	0.0462 (7)	0.0551 (8)	−0.0017 (6)	0.0021 (6)	−0.0027 (6)
P1	0.0402 (7)	0.0533 (7)	0.0360 (7)	−0.0027 (6)	−0.0063 (5)	0.0034 (6)
P2	0.0383 (7)	0.0454 (7)	0.0363 (7)	−0.0003 (5)	−0.0036 (5)	0.0033 (5)
O1	0.119 (3)	0.070 (3)	0.117 (4)	−0.039 (2)	0.041 (3)	−0.014 (2)
O2	0.143 (4)	0.122 (4)	0.048 (3)	0.005 (3)	0.019 (2)	−0.002 (2)
O3	0.130 (4)	0.040 (2)	0.147 (4)	0.002 (2)	0.020 (3)	−0.012 (2)
O4	0.104 (3)	0.131 (4)	0.111 (4)	0.025 (3)	−0.029 (3)	−0.001 (3)
O5	0.121 (4)	0.120 (4)	0.116 (4)	−0.037 (3)	0.068 (3)	−0.067 (3)
C1	0.048 (3)	0.051 (3)	0.033 (3)	−0.005 (2)	−0.001 (2)	0.001 (2)
C2	0.053 (3)	0.073 (3)	0.052 (3)	0.001 (3)	−0.004 (2)	0.014 (3)
C3	0.075 (4)	0.071 (4)	0.057 (4)	0.017 (3)	−0.011 (3)	0.009 (3)
C4	0.111 (5)	0.059 (3)	0.047 (4)	0.017 (3)	0.003 (3)	0.009 (3)
C5	0.088 (4)	0.067 (4)	0.067 (4)	−0.012 (3)	0.023 (3)	0.011 (3)
C6	0.059 (3)	0.060 (3)	0.051 (3)	−0.003 (3)	0.002 (2)	0.004 (3)
C7	0.035 (2)	0.052 (3)	0.051 (3)	−0.001 (2)	−0.001 (2)	−0.002 (2)
C8	0.092 (4)	0.064 (4)	0.056 (4)	0.004 (3)	−0.018 (3)	−0.010 (3)
C9	0.135 (6)	0.076 (5)	0.077 (5)	0.006 (4)	−0.031 (4)	−0.027 (4)
C10	0.081 (4)	0.059 (4)	0.111 (6)	0.003 (3)	−0.007 (4)	−0.024 (4)
C11	0.089 (4)	0.060 (4)	0.087 (5)	−0.001 (3)	0.009 (3)	0.003 (3)
C12	0.082 (4)	0.063 (4)	0.049 (3)	−0.006 (3)	−0.003 (3)	−0.001 (3)
C13	0.047 (3)	0.047 (3)	0.035 (3)	0.000 (2)	−0.004 (2)	0.003 (2)
C14	0.058 (3)	0.061 (3)	0.060 (4)	−0.001 (3)	0.000 (3)	−0.007 (3)
C15	0.082 (4)	0.066 (4)	0.070 (4)	0.006 (3)	0.010 (3)	−0.017 (3)
C16	0.075 (4)	0.065 (4)	0.065 (4)	0.015 (3)	0.023 (3)	0.005 (3)
C17	0.058 (3)	0.076 (4)	0.068 (4)	−0.005 (3)	0.018 (3)	0.006 (3)
C18	0.059 (3)	0.062 (3)	0.048 (3)	−0.007 (3)	0.004 (2)	−0.007 (2)
C19	0.041 (3)	0.052 (3)	0.040 (3)	−0.002 (2)	−0.002 (2)	−0.002 (2)
C20	0.066 (3)	0.057 (3)	0.055 (4)	−0.007 (2)	0.003 (2)	−0.007 (3)
C21	0.075 (4)	0.072 (4)	0.075 (4)	−0.002 (3)	0.007 (3)	−0.033 (3)
C22	0.082 (4)	0.051 (4)	0.112 (6)	−0.008 (3)	0.011 (4)	−0.014 (4)
C23	0.128 (6)	0.055 (4)	0.090 (5)	−0.018 (3)	0.030 (4)	0.004 (4)
C24	0.101 (4)	0.054 (3)	0.056 (4)	−0.007 (3)	0.017 (3)	−0.002 (3)
C25	0.038 (3)	0.043 (3)	0.038 (3)	0.000 (2)	0.0021 (19)	0.001 (2)
C26	0.051 (3)	0.051 (3)	0.060 (3)	0.000 (2)	−0.009 (2)	0.009 (2)
C27	0.046 (3)	0.072 (4)	0.065 (4)	0.008 (3)	−0.009 (2)	0.014 (3)
C28	0.038 (3)	0.090 (4)	0.081 (4)	−0.002 (3)	−0.007 (3)	0.000 (3)
C29	0.054 (4)	0.058 (4)	0.170 (7)	−0.018 (3)	−0.011 (4)	0.007 (4)
C30	0.053 (3)	0.050 (3)	0.117 (5)	−0.001 (3)	−0.010 (3)	0.015 (3)
C31	0.043 (3)	0.042 (3)	0.044 (3)	−0.002 (2)	0.002 (2)	−0.003 (2)
C32	0.056 (3)	0.103 (4)	0.049 (4)	0.019 (3)	−0.007 (3)	−0.014 (3)
C33	0.082 (5)	0.129 (6)	0.048 (4)	0.017 (4)	0.002 (3)	−0.020 (4)
C34	0.090 (5)	0.074 (4)	0.064 (4)	−0.009 (3)	0.032 (3)	−0.019 (3)
C35	0.048 (3)	0.088 (4)	0.092 (5)	−0.004 (3)	0.015 (3)	−0.026 (4)
C36	0.044 (3)	0.082 (4)	0.063 (4)	−0.003 (3)	0.000 (3)	−0.016 (3)
C37	0.096 (5)	0.095 (5)	0.110 (6)	0.016 (4)	0.032 (4)	−0.003 (4)

### Geometric parameters (Å, °)

**Table:**

Ag1—P1	2.4309 (12)	C27—C28	1.343 (8)
Ag1—P2	2.4277 (12)	C28—C29	1.359 (8)
Ag1—O1	2.540 (4)	C29—C30	1.369 (8)
Ag1—O5	2.413 (5)	C31—C36	1.371 (6)
Cl1—O1	1.413 (4)	C31—C32	1.365 (7)
Cl1—O2	1.408 (4)	C32—C33	1.381 (8)
Cl1—O3	1.407 (4)	C33—C34	1.348 (10)
Cl1—O4	1.407 (4)	C34—C35	1.366 (8)
P1—C1	1.819 (4)	C35—C36	1.374 (8)
P1—C7	1.815 (4)	C2—H2	0.9300
P1—C13	1.818 (4)	C3—H3	0.9300
P2—C19	1.811 (4)	C4—H4	0.9300
P2—C25	1.821 (4)	C5—H5A	0.9300
P2—C31	1.821 (4)	C6—H6	0.9300
O5—C37	1.378 (9)	C8—H8	0.9300
O5—H5	0.8200	C9—H9	0.9300
C1—C2	1.388 (6)	C10—H10	0.9300
C1—C6	1.379 (7)	C11—H11	0.9300
C2—C3	1.371 (7)	C12—H12	0.9300
C3—C4	1.356 (8)	C14—H14	0.9300
C4—C5	1.360 (9)	C15—H15	0.9300
C5—C6	1.377 (7)	C16—H16	0.9300
C7—C12	1.382 (7)	C17—H17	0.9300
C7—C8	1.368 (7)	C18—H18	0.9300
C8—C9	1.385 (8)	C20—H20	0.9300
C9—C10	1.362 (9)	C21—H21	0.9300
C10—C11	1.358 (10)	C22—H22	0.9300
C11—C12	1.365 (7)	C23—H23	0.9300
C13—C14	1.387 (7)	C24—H24	0.9300
C13—C18	1.377 (6)	C26—H26	0.9300
C14—C15	1.376 (8)	C27—H27	0.9300
C15—C16	1.365 (8)	C28—H28	0.9300
C16—C17	1.360 (8)	C29—H29	0.9300
C17—C18	1.379 (7)	C30—H30	0.9300
C19—C24	1.373 (7)	C32—H32	0.9300
C19—C20	1.378 (7)	C33—H33	0.9300
C20—C21	1.384 (7)	C34—H34	0.9300
C21—C22	1.365 (9)	C35—H35	0.9300
C22—C23	1.354 (9)	C36—H36	0.9300
C23—C24	1.386 (7)	C37—H37A	0.9600
C25—C26	1.370 (6)	C37—H37B	0.9600
C25—C30	1.366 (7)	C37—H37C	0.9600
C26—C27	1.374 (6)		
			
P1—Ag1—P2	133.15 (4)	C31—C32—C33	120.8 (5)
P1—Ag1—O1	97.68 (10)	C32—C33—C34	120.7 (6)
P1—Ag1—O5	107.44 (12)	C33—C34—C35	119.5 (6)
P2—Ag1—O1	114.20 (9)	C34—C35—C36	119.7 (5)
P2—Ag1—O5	104.75 (11)	C31—C36—C35	121.5 (5)
O1—Ag1—O5	92.22 (13)	C1—C2—H2	120.00
O1—Cl1—O2	110.3 (2)	C3—C2—H2	120.00
O1—Cl1—O3	109.3 (2)	C2—C3—H3	120.00
O1—Cl1—O4	108.1 (3)	C4—C3—H3	120.00
O2—Cl1—O3	110.6 (2)	C3—C4—H4	120.00
O2—Cl1—O4	109.8 (2)	C5—C4—H4	120.00
O3—Cl1—O4	108.8 (3)	C4—C5—H5A	120.00
Ag1—P1—C1	119.74 (14)	C6—C5—H5A	119.00
Ag1—P1—C7	109.89 (14)	C1—C6—H6	120.00
Ag1—P1—C13	112.05 (14)	C5—C6—H6	120.00
C1—P1—C7	107.36 (18)	C7—C8—H8	120.00
C1—P1—C13	102.22 (19)	C9—C8—H8	120.00
C7—P1—C13	104.31 (19)	C8—C9—H9	120.00
Ag1—P2—C19	110.32 (14)	C10—C9—H9	120.00
Ag1—P2—C25	117.97 (13)	C9—C10—H10	120.00
Ag1—P2—C31	115.11 (14)	C11—C10—H10	120.00
C19—P2—C25	103.04 (19)	C10—C11—H11	120.00
C19—P2—C31	104.97 (18)	C12—C11—H11	120.00
C25—P2—C31	104.01 (19)	C7—C12—H12	119.00
Ag1—O1—Cl1	133.7 (2)	C11—C12—H12	119.00
Ag1—O5—C37	129.5 (3)	C13—C14—H14	119.00
C37—O5—H5	109.00	C15—C14—H14	120.00
Ag1—O5—H5	118.00	C14—C15—H15	120.00
P1—C1—C6	118.9 (3)	C16—C15—H15	120.00
P1—C1—C2	122.6 (3)	C15—C16—H16	120.00
C2—C1—C6	118.4 (4)	C17—C16—H16	120.00
C1—C2—C3	120.4 (4)	C16—C17—H17	120.00
C2—C3—C4	120.7 (5)	C18—C17—H17	120.00
C3—C4—C5	119.5 (5)	C13—C18—H18	120.00
C4—C5—C6	121.1 (5)	C17—C18—H18	120.00
C1—C6—C5	119.8 (5)	C19—C20—H20	120.00
P1—C7—C12	117.9 (3)	C21—C20—H20	119.00
P1—C7—C8	124.1 (3)	C20—C21—H21	120.00
C8—C7—C12	117.9 (4)	C22—C21—H21	120.00
C7—C8—C9	120.4 (5)	C21—C22—H22	120.00
C8—C9—C10	120.5 (6)	C23—C22—H22	120.00
C9—C10—C11	119.5 (6)	C22—C23—H23	120.00
C10—C11—C12	120.1 (5)	C24—C23—H23	120.00
C7—C12—C11	121.6 (5)	C19—C24—H24	120.00
P1—C13—C14	117.5 (3)	C23—C24—H24	120.00
C14—C13—C18	117.8 (4)	C25—C26—H26	119.00
P1—C13—C18	124.5 (3)	C27—C26—H26	119.00
C13—C14—C15	121.1 (5)	C26—C27—H27	120.00
C14—C15—C16	119.9 (5)	C28—C27—H27	120.00
C15—C16—C17	119.9 (5)	C27—C28—H28	120.00
C16—C17—C18	120.5 (5)	C29—C28—H28	120.00
C13—C18—C17	120.7 (4)	C28—C29—H29	119.00
P2—C19—C24	123.3 (3)	C30—C29—H29	120.00
P2—C19—C20	118.2 (3)	C25—C30—H30	120.00
C20—C19—C24	118.5 (4)	C29—C30—H30	120.00
C19—C20—C21	121.0 (5)	C31—C32—H32	120.00
C20—C21—C22	119.3 (5)	C33—C32—H32	120.00
C21—C22—C23	120.5 (5)	C32—C33—H33	120.00
C22—C23—C24	120.4 (5)	C34—C33—H33	120.00
C19—C24—C23	120.3 (5)	C33—C34—H34	120.00
P2—C25—C26	123.9 (3)	C35—C34—H34	120.00
C26—C25—C30	117.7 (4)	C34—C35—H35	120.00
P2—C25—C30	118.4 (3)	C36—C35—H35	120.00
C25—C26—C27	121.3 (5)	C31—C36—H36	119.00
C26—C27—C28	120.3 (5)	C35—C36—H36	119.00
C27—C28—C29	119.2 (5)	O5—C37—H37A	109.00
C28—C29—C30	121.0 (5)	O5—C37—H37B	109.00
C25—C30—C29	120.5 (5)	O5—C37—H37C	109.00
P2—C31—C32	122.8 (4)	H37A—C37—H37B	110.00
C32—C31—C36	117.8 (4)	H37A—C37—H37C	109.00
P2—C31—C36	119.4 (4)	H37B—C37—H37C	110.00
			
P2—Ag1—P1—C1	−169.50 (15)	C31—P2—C25—C30	84.1 (4)
P2—Ag1—P1—C7	65.60 (15)	Ag1—P2—C31—C32	142.6 (3)
P2—Ag1—P1—C13	−49.87 (15)	Ag1—P2—C31—C36	−35.9 (4)
O1—Ag1—P1—C1	−35.61 (18)	C19—P2—C31—C32	−95.9 (4)
O1—Ag1—P1—C7	−160.50 (17)	C19—P2—C31—C36	85.6 (4)
O1—Ag1—P1—C13	84.02 (17)	C25—P2—C31—C32	12.0 (4)
O5—Ag1—P1—C1	59.18 (18)	C25—P2—C31—C36	−166.5 (4)
O5—Ag1—P1—C7	−65.72 (17)	P1—C1—C2—C3	−175.7 (4)
O5—Ag1—P1—C13	178.80 (17)	C6—C1—C2—C3	0.6 (7)
P1—Ag1—P2—C19	−56.81 (16)	P1—C1—C6—C5	175.6 (4)
P1—Ag1—P2—C25	−174.79 (15)	C2—C1—C6—C5	−0.8 (7)
P1—Ag1—P2—C31	61.73 (16)	C1—C2—C3—C4	−0.5 (8)
O1—Ag1—P2—C19	174.72 (17)	C2—C3—C4—C5	0.6 (8)
O1—Ag1—P2—C25	56.74 (18)	C3—C4—C5—C6	−0.9 (8)
O1—Ag1—P2—C31	−66.74 (18)	C4—C5—C6—C1	1.0 (8)
O5—Ag1—P2—C19	75.38 (18)	P1—C7—C8—C9	−178.2 (4)
O5—Ag1—P2—C25	−42.60 (19)	C12—C7—C8—C9	−1.4 (8)
O5—Ag1—P2—C31	−166.08 (18)	P1—C7—C12—C11	178.8 (4)
P1—Ag1—O1—Cl1	127.5 (3)	C8—C7—C12—C11	1.9 (8)
P2—Ag1—O1—Cl1	−87.7 (3)	C7—C8—C9—C10	−0.5 (9)
O5—Ag1—O1—Cl1	19.6 (3)	C8—C9—C10—C11	2.1 (9)
P1—Ag1—O5—C37	−156.7 (4)	C9—C10—C11—C12	−1.6 (9)
P2—Ag1—O5—C37	57.9 (5)	C10—C11—C12—C7	−0.4 (8)
O1—Ag1—O5—C37	−57.9 (5)	P1—C13—C14—C15	−177.6 (4)
O2—Cl1—O1—Ag1	−60.3 (4)	C18—C13—C14—C15	−1.3 (7)
O3—Cl1—O1—Ag1	177.9 (3)	P1—C13—C18—C17	177.3 (4)
O4—Cl1—O1—Ag1	59.7 (4)	C14—C13—C18—C17	1.3 (7)
Ag1—P1—C1—C2	158.6 (3)	C13—C14—C15—C16	0.3 (8)
Ag1—P1—C1—C6	−17.7 (4)	C14—C15—C16—C17	0.8 (8)
C7—P1—C1—C2	−75.3 (4)	C15—C16—C17—C18	−0.8 (8)
C7—P1—C1—C6	108.4 (4)	C16—C17—C18—C13	−0.3 (8)
C13—P1—C1—C2	34.1 (4)	P2—C19—C20—C21	−173.3 (4)
C13—P1—C1—C6	−142.2 (3)	C24—C19—C20—C21	3.1 (7)
Ag1—P1—C7—C8	126.0 (4)	P2—C19—C24—C23	173.8 (4)
Ag1—P1—C7—C12	−50.8 (4)	C20—C19—C24—C23	−2.5 (8)
C1—P1—C7—C8	−5.8 (5)	C19—C20—C21—C22	−1.4 (8)
C1—P1—C7—C12	177.5 (4)	C20—C21—C22—C23	−1.1 (9)
C13—P1—C7—C8	−113.7 (4)	C21—C22—C23—C24	1.8 (9)
C13—P1—C7—C12	69.5 (4)	C22—C23—C24—C19	0.1 (9)
Ag1—P1—C13—C14	−41.4 (4)	P2—C25—C26—C27	175.8 (4)
Ag1—P1—C13—C18	142.6 (3)	C30—C25—C26—C27	−2.0 (7)
C1—P1—C13—C14	88.1 (4)	P2—C25—C30—C29	−174.7 (5)
C1—P1—C13—C18	−88.0 (4)	C26—C25—C30—C29	3.2 (8)
C7—P1—C13—C14	−160.2 (4)	C25—C26—C27—C28	−0.7 (8)
C7—P1—C13—C18	23.8 (4)	C26—C27—C28—C29	2.1 (9)
Ag1—P2—C19—C20	−42.0 (4)	C27—C28—C29—C30	−0.8 (10)
Ag1—P2—C19—C24	141.7 (4)	C28—C29—C30—C25	−1.9 (10)
C25—P2—C19—C20	84.8 (4)	P2—C31—C32—C33	−177.1 (4)
C25—P2—C19—C24	−91.5 (4)	C36—C31—C32—C33	1.4 (7)
C31—P2—C19—C20	−166.6 (3)	P2—C31—C36—C35	178.0 (4)
C31—P2—C19—C24	17.1 (4)	C32—C31—C36—C35	−0.6 (7)
Ag1—P2—C25—C26	137.5 (3)	C31—C32—C33—C34	−1.5 (9)
Ag1—P2—C25—C30	−44.7 (4)	C32—C33—C34—C35	0.6 (9)
C19—P2—C25—C26	15.7 (4)	C33—C34—C35—C36	0.2 (8)
C19—P2—C25—C30	−166.5 (4)	C34—C35—C36—C31	−0.2 (8)
C31—P2—C25—C26	−93.6 (4)		

### Hydrogen-bond geometry (Å, °)

**Table:**

*D*—H···*A*	*D*—H	H···*A*	*D*···*A*	*D*—H···*A*
O5—H5···O2^i^	0.82	2.42	3.157 (6)	151
O5—H5···O3^i^	0.82	2.30	3.033 (6)	150

Symmetry codes: (i) −*x*+1/2, *y*+1/2, −*z*+1/2.
